# Evaluating the effectiveness of a support programme for people with type 2 diabetes mellitus in primary care: an observational prospective study in Ecuador

**DOI:** 10.3399/bjgpopen20X101025

**Published:** 2020-04-22

**Authors:** Francisco Barrera-Guarderas, Katherine De la Torre-Cisneros, Maria Barrionuevo-Tapia, Carmen Cabezas-Escobar

**Affiliations:** 1 College of Medicine, Pontifical Catholic University of Ecuador, Quito, Ecuador; 2 College of Medical Sciences, State University of Southern Manabi, Jipijapa, Ecuador; 3 College of Medicine, Pontifical Catholic University of Ecuador, Quito, Ecuador; 4 College of Medicine, Pontifical Catholic University of Ecuador, Quito, Ecuador

**Keywords:** diabetes mellitus, primary health care, non-communicable diseases, health systems

## Abstract

**Background:**

The success of primary health care relies on the integration of empowered practitioners with cooperative patients regardless of socioeconomic status. Using resources efficiently would help to improve healthcare promotion and reduce complications of chronic non-communicable diseases (NCDs). The importance of network support programmes relies on the fact that they allow to accurately deliver medical care by shaping a sense of community and purpose among the patients.

**Aim:**

To evaluate the effectiveness of a network support programme for patients with type 2 diabetes mellitus (T2DM).

**Design & setting:**

A centre-based observational prospective study took place in a primary care setting in Ecuador.

**Method:**

The impact of the diabetes care programme was assessed by comparing initial and final metabolic characteristics and outcomes of 593 patients with T2DM, followed-up from April 2007 to December 2017, using paired sample *t*-test. Electrocardiograms (ECGs), ankle-brachial indexes (ABIs), ocular fundus, and monofilament neuropathy tests were assessed with the McNemar test to evaluate complications at the beginning and end of the study.

**Results:**

Glycated haemoglobin (HbA1c), lipid profile, and systolic blood pressure (SBP) showed statistically significant decreases between the initial measurement (IMs) and final measurements (FMs). In the FM, significantly lower HbA1c, diastolic blood pressure (DBP), and atherogenic index were found. Despite the length of time since diagnosis, during the follow-up time, long-term micro- and macro-vascular complications, such as ocular fundus, serum creatinine, and ABI, remained unchanged throughout the period of active participation in this healthcare programme.

**Conclusion:**

This study demonstrates the feasibility of reducing plasma glucose, plasma lipids, and long-term complications in patients with T2DM by implementing a network support programme, which involves the medical team and patients themselves in an environment with limited resources.

## How this fits in

There are successful medical care models worldwide; however, in Latin American countries, application of these models is difficult to fulfil owing to the lack of public policies. This study shows that a comprehensive intervention can be beneficial in the medium- and long-term prevention of diabetes complications when delivered in primary health care in developing countries.

## Introduction

The prevalence of T2DM, initially considered a disease of high-income countries, has become a significant public health problem increasingly affecting low- and middle-income countries.^1^ It is predicted that 550 million people will have T2DM by 2030, an increase of 50% compared with current data.^2^ In 2017, 8% of the adult population of Central and South America was estimated to suffer from diabetes; in Ecuador, it is estimated that 5.5% (3.5%–8.5%) of the population aged between 20–79 years have T2DM.^3^

The Ecuadorian Constitution protects and implements the right to health. The National Plan for Good Living was developed as a strategy to transform the structure of health care and, therefore, improve the quality of life of the Ecuadorian population. To this end, the Comprehensive Health Care Model (*Modelo de Atención Integral en Salud* [MAIS]) has gradually been implemented in health centres across the country with a focus on chronic diseases.^4^

The Chimbacalle Medical Centre, operated by the Ministry of Public Health of Ecuador, follows the health policy guidelines regarding usual care for patients with T2DM. It consists of a general medical consultation once every 3 months, in which the patient receives general advice and medicine. However, this particular healthcare centre offers a unique additional programme that aims to improve the care of patients with NCDs, specifically patients with T2DM. Patient care was led by one internist and one nurse during the continuity of the entire health programme, and was supported by family medicine rotating-resident physicians. The diabetes care programme strengthens the participation of patients through the mobilisation of a support network called the 'Patients with Diabetes Club'. This organisational model serves to improve the quality of care mostly for people with low-middle incomes, although the service is open to every patient who might need the services provided by the health centre.^5^

The objective of this study was to evaluate the effectiveness of the diabetes care programme for patients with T2DM by comparing the metabolic characteristics of the patients when they entered the programme with their metabolic characteristics after taking part in the programme (April 2007 to December 2017).

## Method

### Setting

The Chimbacalle Medical Centre is a community healthcare centre located 2800 metres above sea level in the metropolitan area of Quito, Ecuador. It is operated by the Ministry of Public Health of Ecuador and provides free laboratory tests, complementary examinations, and medication. It offers health services for men and women with limited economic resources.

### Study design

A centre-based observational prospective study was conducted to evaluate the impact of the comprehensive healthcare approach to T2DM provided by Chimbacalle Medical Centre in Quito, Ecuador.

The diabetes clinic was started to deliver a strategy to fulfil the need to provide integral attention-focused care to the increasing number of patients with T2DM. The programme also incorporated patients' relatives that were diagnosed with T2DM through a research of glucose alterations. Moreover, this unique programme is open to all patients with T2DM, regardless of their personal characteristics. Owing to this, a third source of recruitment consists in the referral of patients (cases with low or high complexity) with a stable condition from other healthcare centres.

Owing to the growth in the number of patients, there are now educational talks every 15 days in the health centre and physical activities, such as dancing and tai chi, take place three times a week. The programme of diabetes care includes cardiovascular^6,7^ and renovascular^8,9^ prophylaxis, lipid-lowering medication,^10,11^ hypoglycants,^12–15^ and antihypertensives medication (Supplementary Table 1).

Study individuals had to have a record of their initial consultation, consisting of an analysis of the history of the disease, its evolution, the pillars of the treatment to be followed by the patient, and the initial indications. Patients had a week to complete individual laboratory tests, ECG, ABI, monofilament test, and oculus fundus before a second consultation. The laboratory tests included blood count, glucose, kidney profile (including albuminuria), liver profile, lipid profile, and glycosylated haemoglobin A1c. These examinations would be repeated at 4–6 months, according to the guidelines for clinical practice.^16^ Afterwards, fasting-glucose monitoring and daily post-prandial assessment would be performed every 2 hours for a week with a capillary glucose test, which allows the medication or insulin dose to be corrected.^17^ Mean plasma levels of lipid profile, HbA1c, urea, and creatinine were measured with a chemistry analyser (Mindray BS-200). SBP, DBP, and body mass index (BMI) were determined for each measurement stage. Atherogenic indexes were calculated using their respective formulas.^18,19^

**Figure 1.**
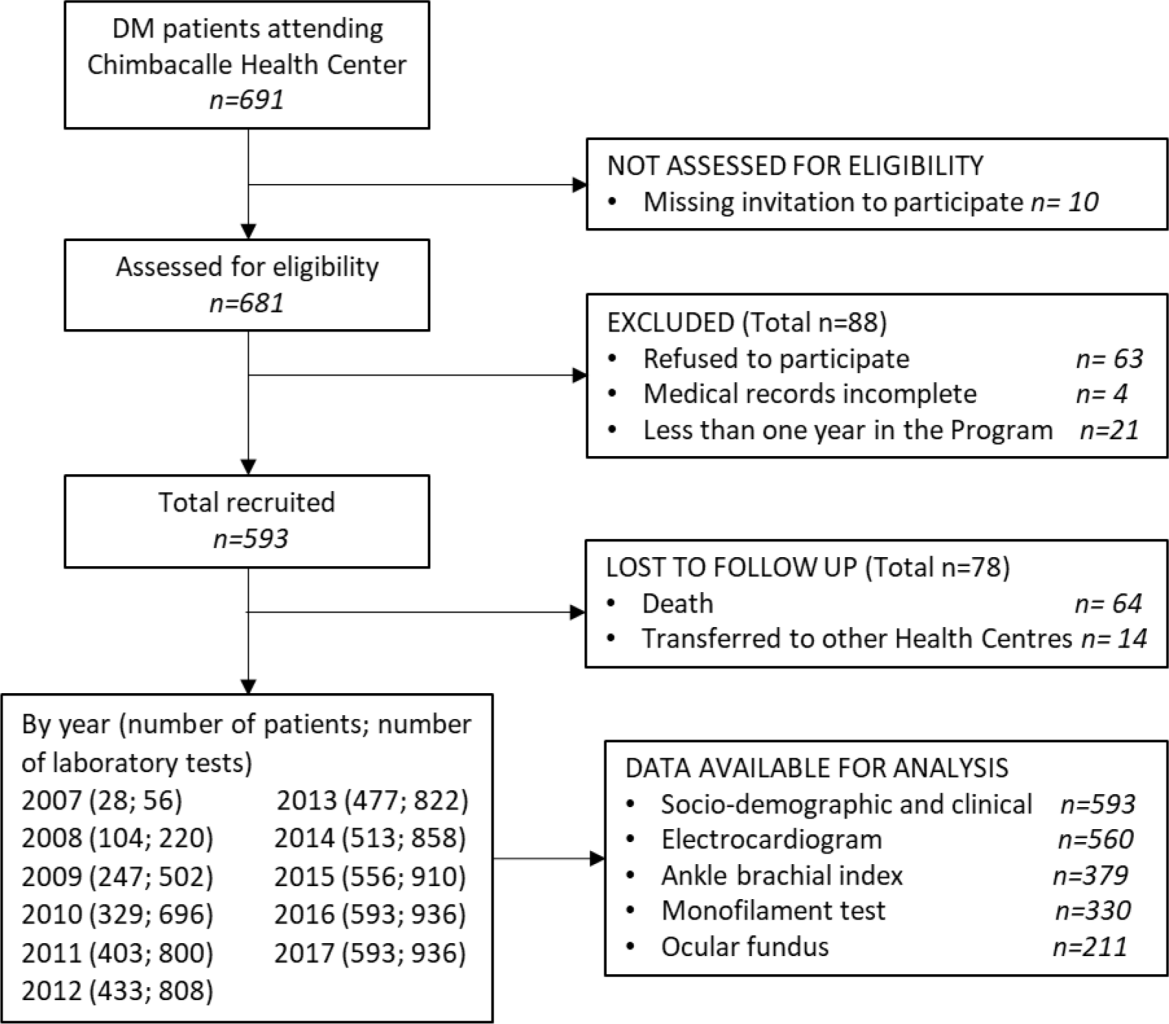
Flow diagram of patient recruitment and follow-up. DM = diabetes mellitus.

Data gathered from the medical records by the medical team included sociodemographic variables and IM and FM metabolic variables in patients with between 1–10 years of treatment at the medical centre. All patients (*n* = 593) who were diagnosed or treated for T2DM at the medical centre for ≥1 year and who had data available for their metabolic features by December 2017 (Figure 1) were included in the study regardless of age, sex, or other medical conditions.

Statistical analysis of the data was performed with IBM SPSS Statistics (version 23). Baseline characteristics were analysed using frequency distribution and central tendency measurements; Mann-Whitney U-test (quantitative variables) and χ^2^ test (categorical variables) were used to estimate differences between sexes. Mean differences were tested using the paired sample *t*-test, which was used to compare metabolic variables across both initial measurements (IMs) and final measurements (FMs). Additionally, variables BMI, HbA1c, SBP, DBP, atherogenic index total cholesterol (TC) and/or high-density lipoprotein (HDL) (high risk of CVD ˃4), and atherogenic index plasma (high risk of CDV ˃0.24) were categorised and analysed by adjusting each of their means to the number of laboratory test results during the period of time in which the patient was actively receiving care through the programme, which only influenced the FM values. Furthermore, McNemar's tests were used to detect the differences in patients' IM and FM ECG, ABI, monofilament neuropathy test, ocular fundus, and categorised HbA1c.

Patients provided written informed consent before being enrolled. The study used a de-identified dataset to protect the confidentiality of the participants.

## Results

The baseline demographic and clinical characteristics of the patients are shown in Table 1. The majority of the participants included were female (*n* = 459, 77.4%). Additionally, 82.1% (*n* = 487) of patients were on simvastatin therapy and 17.0% (*n* = 101) were receiving gemfibrozil (data not shown). The annual attendance compliance ranged from 86.5% to 100%. During the 10 years of follow-up, 64 participants died. All-cause mortality was 10.8% (95% confidence interval [CI] = 8.3 to 13.3) and cumulative incidence of CVD mortality was 2.7% (95% CI = 1.4 to 4.0), representing 25.0% of all deaths.

**Table 1.** Baseline characteristics of patients with type 2 diabetes mellitus.

**Characteristics**	**All,** ***N* =** **593**	**Female,** ***n* =** **459**	**Male,** ***n* =** **134**
Mean age, years (SD)	66.5 (12.3)	66.5 (12.0)	66.5 (13.5)
Mean age at diagnosis, years (SD)	52.7 (12.2)	52.3 (11.8)	53.8(13.7)
Mean time of disease, years (SD)*	13.8 (7.9)	14.2 (8.0)	13.0 (7.4)
Mixed race, *n* (%)	588 (99.2)	456 (77.6)	132 (22.4)
Hypertension, *n* (%)	387 (65.3)	306 (79.1)	81 (20.9)
Family history of CVD, *n* (%)	343 (57.8)	262 (76.4)	81 (23.6)
**Tobacco use** *		
Non-smokers, *n* (%)	464 (78.2)	404 (87.1)	60 (12.9)
Former Smokers, *n* (%)	83 (14.0)	40 (48.2)	43 (51.8)
Smokers, *n* (%)	46 (7.8)	15 (32.6)	31 (67.4)
Mean programme engagement time, years (SD)*	5.5 (2.7)	5.7 (2.6)	4.9 (2.7)

CVD = cardiovascular disease. SD = standard deviation.

*Time of disease, programme engagement time, and tobacco use were significantly different among the sexes (Mann-Whitney U-test, χ^2^ test; statistically significant *P*<0.05).

All metabolic variables of interest are reported in Table 2, showing all IMs and FMs for average BMI, SBP, DBP, HbA1c, lipid profile, atherogenic indexes, urea, and creatinine during the follow-up period. There was an overall reduction of patient’s weight; however, this loss was minimal and patients continued to be characterised as overweight. Although all final metabolic variable measurements, including HbA1c, showed mean differences comparing the IM and FMs, no significant differences in average SBP, urea, and creatinine were found. Regardless of the duration of illness, during follow-up no deterioration was determined in the renal function of patients.

**Table 2.** Mean difference between initial and final measurements for clinical features and metabolic variables in patients with type 2 diabetes mellitus.

**Metabolic variables**	**Measurements**	
**Initial,** ***n* =** **593**	**Final,** ***n* =** **593**	**Mean difference**	*P*-value*
**Mean**	**SD**	**Mean**	**SD**
**BMI**	29.32	4.94	29.06	4.61	–0.26	0.013
**SBP**	124.24	18.35	122.63	16.82	–1.61	0.054
**DBP**	75.22	11.08	71.84	9.13	3.37	<0.01
**HbA1c**	8.29	2.39	7.83	1.65	0.46	<0.01
**TC**	206.29	49.82	184.56	45.78	21.73	<0.01
**HDL-C**	54.96	17.59	59.63	14.73	–4.67	<0.01
**LDL-C**	113.34	44.72	92.32	35.72	21.02	<0.01
**Triglycerides**	190.98	123.74	163.01	91.33	27.97	<0.01
**Urea**	40.10	15.82	41.16	23.81	–1.05	0.272
**Creatinine**	1.06	0.33	1.11	0.60	–0.04	0.063
**TC/HDL-C**	4.08	1.56	3.19	0.83	0.89	<0.01
**LDL/HDL-C**	2.29	1.23	1.60	0.64	0.69	<0.01
**TC-HDL-C**	151.32	49.44	124.92	41.78	26.40	<0.01
**TC-HDL-C/HDL-C**	3.08	1.56	2.19	0.83	0.89	<0.01
**TG/HDL-C**	3.83	2.63	2.95	2.19	0.88	<0.01
**AIP**	0.14	0.27	0.04	0.24	0.10	<0.01

AIP = atherogenic index plasma. BMI = body mass index. DBP = diastolic blood pressure. HbA1c = glycated haemoglobin. HDL-C = high-density lipoprotein cholesterol. LDL-C = low-density lipoprotein cholesterol. SBP = systolic blood pressure. SD = standard deviation. TC = total cholesterol. TG = triglyceride.

*Paired samples *t*-test; statistically significant *P* ≤0.05.

Additionally, 54.5% of the patients' HbA1c values were <8.0% in their IM; however, at the time of the FM, this percentage increased to 62.6% of the patients portraying HbA1c values <8.0%. This shows a significant improvement (*P*<0.001) in diabetes control as the number of patients with HbA1c values of <8.0% increased throughout the programme.

**Figure 2.**
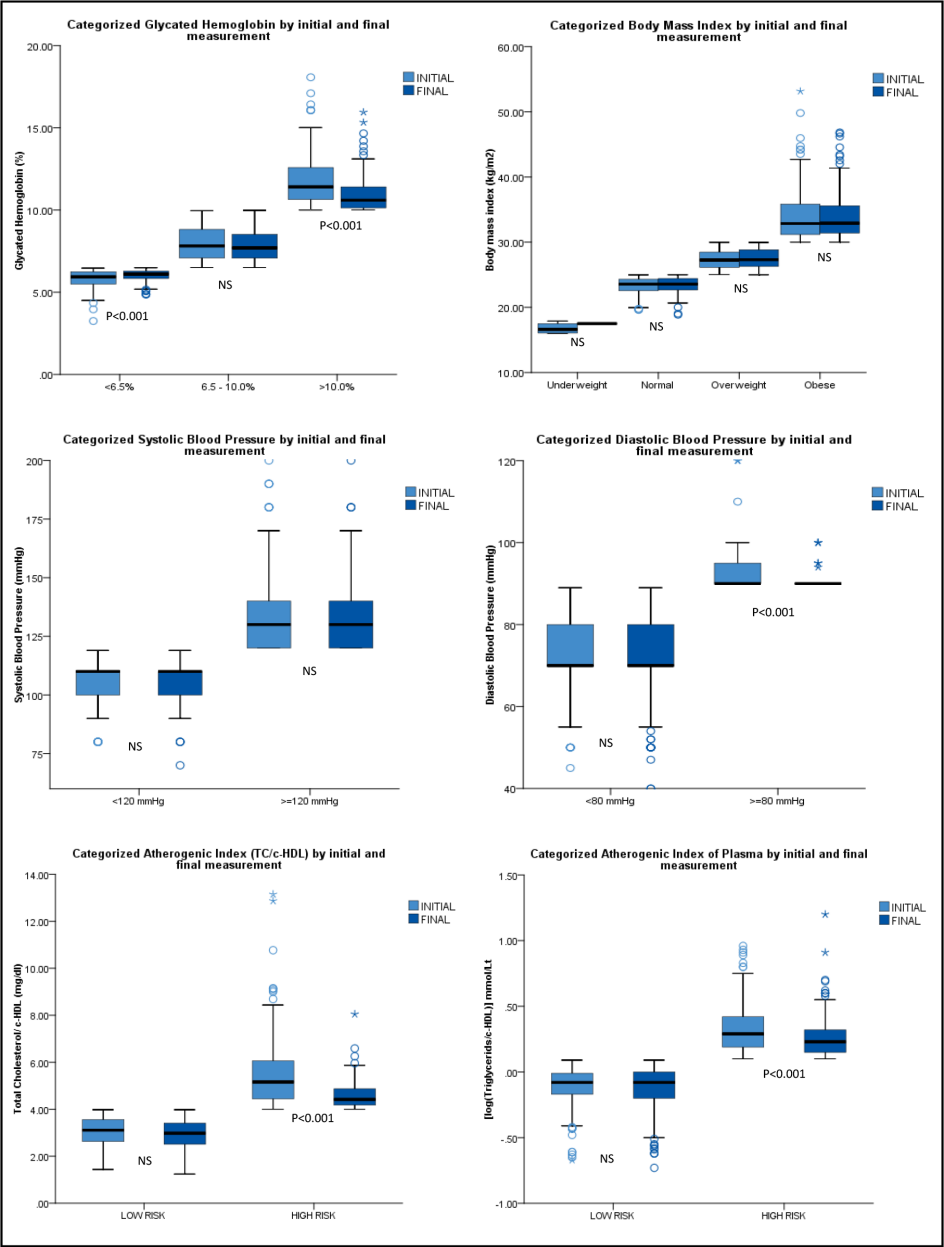
Metabolic variables categorised according to the initial and final measurement. Paired samples *t*-test; statistically significant *P*≤0.05. HDL-C = high-density lipoprotein cholesterol. NS = non-significant. TC = total cholesterol.

Figure 2 shows a comparison between the IMs and FMs of control by categorised metabolic variables. It was observed that the difference between categorised HbA1c groups, DBP (≥80 mmHg), atherogenic index (TC and/or HDL-cholesterol [c]) high-risk group, and atherogenic plasma index high-risk group were statistically significant (*P*<0.001) among IMs and FMs. In groups categorised for HbA1c, people with HbA1c levels >10.0% showed decreases of 3.6% (95% CI = 3.5 to 3.8) compared with the IM. On the other hand, people with HbA1c <6.5% had a slight increase of 1.4% (95% CI = 1.3 to 1.6), with their HbA1c value remaining within the adequate range for patients with T2DM.

According to the results summarised in Table 3, there was a statistically significant increase of 6.9% (*P*<0.01) in the number of patients who had an abnormal ECG, from the IM (59.5%) to the FM (66.4%). The IM of monofilament neuropathy test increased significantly (*P*<0.01) in the FM (4.5%), suggesting a probable increasing relation with the years of duration of diabetes. The findings for ABI and ocular fundus were statistically non-significant.

**Table 3.** Electrocardiogram, ankle-brachial index, monofilament neuropathy test, and ocular fundus by initial and final measurement in patients with type 2 diabetes mellitus.

	**Measurement**	
**Examinations**	**Initial**		**Final**		*P*-value*
	***n***	**%**	***n***	**%**	
**ECG (*n* = 560**)					
Normal	227	40.5	188	33.6	0.005
Abnormal	333	59.5	372	66.4	
**ABI (*n* = 379**)					
Normal	330	87.1	321	84.7	0.298
Abnormal	49	12.9	58	15.3	
**Monofilament (*n* = 330**)					
Normal	313	94.8	298	90.3	0.001
Abnormal	17	5.2	32	9.7	
**Ocular fundus (*n* = 211**)					
Normal	182	86.3	191	90.5	0.093
Abnormal	29	13.7	20	9.5	

ABI = ankle-brachial index. ECG = electrocardiogram.

*Related-samples McNemar test; statistically significant *P*≤0.05

## Discussion

### ​Summary

In this group of patients with T2DM, a comprehensive healthcare model was implemented. This was based on the original model proposed by the World Health Organization and the Pan American Health Organization.^20^ The MAIS for NCDs, with adjusted strategies to the care model, obtained favourable responses in participating patients over the 10-year duration of the study.

### ​Strengths and limitations

The success of the Chimbacalle diabetes programme is owing to many factors, but the most important one is the involvement of patients in their own health care. Each patient has their own printed record containing all of their laboratory, clinical, and anthropometric information; this strategy gives patients control over their own health and produces high attendance compliance. Furthermore, the feeling of belonging to a group of peers is transformed into motivation for the adherence to treatment, as well as in the prevention (patients become promoters of health within their family) and control of the disease.^21^

This study is a robust design, but its limitation was the loss of patients that could be a source of bias and a possible overvaluation of strength. Another limitation is the lack of sources of comparison. Given that almost every diagnosed patient in the centre joins the programme, the results cannot be compared with usual healthcare receivers in the same centre. Furthermore, the database of this study is quite unique and there is no similar information in other healthcare centres; however, this study introduces a protocol that can potentially improve patient management, and shows the excellent results of an integral diabetes programme with pharmacological and non-pharmacological intervention strategies that have contributed to public health.

### ​Comparison with existing literature

This research uses an indicator of patient monitoring, metabolic control, and HbA1c. The study findings showed a decrease in HbA1c from 8.3% to 7.8%; it is important to consider that the applied model does not correspond to improved control. When HbA1c was reviewed and analysed in the ADVANCE, ACCORD, and VADT studies, reduction in HbA1c with conventional therapy was 1.0%, 0.6%, and 1.o%, respectively, which does not significantly differ from the population studied here. However, any reduction in HbA1c significantly reduces micro- and macrovascular complications.^10,22,23^

The general reduction of 0.5% in HbA1c found in this study was maintained during the 10-year follow-up, as found in the UK Prospective Diabetes Study (UKPDS), where a 1% reduction in HbA1c was associated with a 37% decrease in microvascular risk.^24^ There are also some findings from Latin America, such as Chilean^25^ and Mexican^26^ studies, with a decrease of 0.4%–0.6% in the intervention group;^27^ and, Colombian^28^ and Peruvian^29^ studies showed a reduction of 1.3% up to 1.8% in the first year and no significant changes in the 4-year follow-up. Other studies around the world indicate that intervention reduces Hb1Ac by about 0.46%.^30^

The prevalence of micro- and macrovascular complications in the study is lower than that reported in the literature,^31^ owing to early onset of pharmacological treatment with metformin at medium and high doses.^32^ Also, patients displaying inadequate response (HbA1c >8.5%) to oral hypoglycaemic drugs were started with Neutral Protamine Hagedorn (NPH) insulin therapy as soon as possible. Renoprotection using renin-angiotensin-aldosterone system blockers at minimum doses was given to patients with ≥5 years of illness or when albuminuria is demonstrated.^8,9^ Furthermore, the stabilisation of lipid profiles took into account atherogenic-risk calculation and, therefore, the start of statins^33^ and the administration of acetylsalicylic acid (ASA) at low doses was based on the cardiovascular-risk score in people with diabetes.^34,35^ Even though there is controversy surrounding the daily use of ASA owing to the blood-thinning effect of aspirin,^36^ no side effects of bleeding have been recorded in these patients. Perhaps, the rate of complications and CVD mortality is extremely lower than other cohorts;^37^ further research is needed.

### ​Implications for practice

Continuing education about diabetes is an essential component in prevention and treatment strategies. The educational strategy combined with clinical treatment provides the necessary incentives to face major changes in the patient's lifestyle.^38^ It is evident that the improvement in diabetes outcomes is linked to many factors; however, patient involvement in their own health care has been identified as a key factor in improving metabolic control of T2DM and delaying the appearance and progression of complications.^39^

An important aspect of this model of care is the permanence and continuity of the health personnel caring for patients. The same doctor and nurse have remained in the health team for 10 years. This is likely to have helped patients’ engagement in the programme and the integration of the medical personnel in all patient contexts.

The initial diagnosis of diabetes is very stressful for patients. The initial shock and fear of this new diagnosis is a major barrier to understanding the indications given by the doctor. The healthcare staff help to resolve questions generated in the process of learning about the disease. Likewise, the staff create links between the medical team, the patient, and their family environment, creating a climate of trust that allows for better follow-up.

The country's health system guarantees the delivery of medication free of charge. It is observed that this could be considered a strategy in the appropriate metabolic control of patients. Adherence to treatment increases when distribution is free, and when laboratory and complementary exams are carried out continuously.^40^ However, treatment is not sufficient without peer support and regular medical care.

In conclusion, Chimbacalle Medical Centre has successfully implemented a state-of-the-art programme that provides patients with T2DM with integral attention-focused care and a support network. By accurately complying with protocols, as well as international and national guidelines, the centre's programme has been useful in achieving adequate metabolic control and reduction of micro- and macrovascular complications. The unique nature of this programme has posed, to a certain extent, a limitation to this study, as a comparable database within the country is non-existent. However, the research presented has portrayed the achievement of positive outcomes, especially in the prevention of complications, despite the fact that the programme takes place in a primary healthcare centre that works with limited resources.
